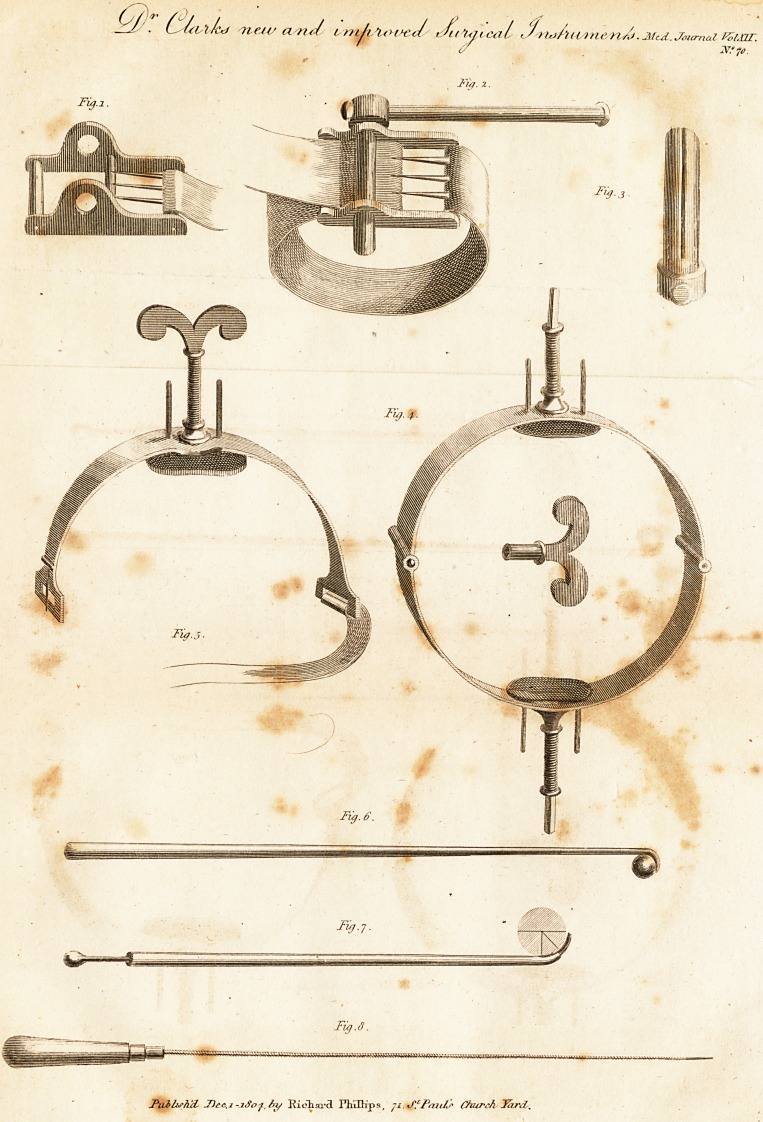# Dr. Clark's Improved Surgical Instruments

**Published:** 1804-12-01

**Authors:** Thomas Clark

**Affiliations:** Verdun sur Meuse


### To Dr. BATTY.

Dear Sir,

When I last had the pleasure of seeing you in London, I promised to give you an account of any surgical instruments that I might be fortunate enough to invent or improve. In the mean time, I enclose drawings of several, with short descriptions of them. In case you are of opinion that that they are likely to prove useful, I will thank you to make them publicly known through the medium of the Medical Journal.

Yours, &c.

*Verdun sur Meuse, May* 30, 1804. THOMAS CLARK, M. D.

---

The tourniquet represented in the drawings, fig. 1, 2, and 3, is much more simple, smaller, lighter, and equally efficacious with any hitherto invented, and may be procured at a very trifling expence. In fact, it furnishes a means of easily tightening a piece of tape or leather strap with a lever. The buckle and tape, represented in fig. 1, being applied to a limb in the common way, the loose portion of tape, next the tongue of the buckle, is to be drawn somewhat tight, and placed with its edges opposed to the centre of the holes of the instrument, as represented in fig. 2. The shortest portion of the lever is now to be introduced into one of the holes, so that its slit, represented in fig. 3, may receive the edge of the tape; then push it along until its extremity passes through the hole in the opposite side of the instrument. The tongue of the buckle being now disengaged from the tape, the lever is to be turned round by means of its movable handle, (a) in the same direction with the points of the tongue of the buckle. When the tape is sufficiently tight, the lever may either be allowed to remain or taken away, as may be found most convenient. By turning it once round in the opposite direction, it can easily be withdrawn. Consequently, this tourniquet is peculiarly well adapted for the army, as, during an engagement, one lever will serve as many tourniquets as an individual can apply. Hence the carrying a considerable number of these instruments becomes a very easy matter, their weight and size being thus rendered very trifling.

Being of opinion that an instrument capable of compressing any of the larger blood vessels of the extremities, while, at the same time, the blood should be permitted to circulate in the parts beyond the instrument, by means of the other vessels, might, on many occasions, prove extremely servicable, I have invented the instrument represented in fig. 4.

Let it be supposed, that the humeral artery is wounded at the flexure of the fore arm, by a lancet in the operation of of blood-letting. In this case, it appears to me more than probable, that if the artery was soon afterwards compressed, at a little distance above the wound, and kept so for several days, the circulation in the smaller arteries being in the mean time in a great measure uninterrupted, that the wound in the artery would heal. When it should be deemed necessary to remove the compression, I would recommend doing it in a very gradual manner. Thus, for the first day, let the compression be so regulated as to permit a fourth part of the usual quantity of blood to pass under the cushion; on the second day, a third part, and so on, provided there was reason to believe that the wound in the artery had perfectly united; if not, the entire compression must necessarily be again had recourse to.

The instrument represented in fig. 4, seems to be well adapted for compressing the humeral artery, or any other that can be easily pressed against a bone. It consists of a semi-circular portion of steel, (the diameter of which should always be greater than that of the limb to which it is to be applied) a screw with a soft cushion attached to it, a piece of tape, and a buckle. The tape ought to be a good deal broader than common, or a large cushion, fixed to a thin plate of metal, should be applied diametrically opposite to the screw, and retained in its place by the tape.

The cushion being applied, by means of the screw, to the internal plate of the instrument, let the semi-circle be placed on the arm, so that the cushion may be immediately over the artery; then buckle the tape with considerable tightness. The screw is now to be turned gradually round, until the circulation in the artery is stopped. It must evidently appear, as the semi-circle does not touch the arm, that the circulation under it, except where the cushion presses, will be totally uninterrupted. However, if the cushion is only applied with such a degree of force as is just sufficient to stop the circulation in the artery, I think it must also be granted, that the circulation under the tape or opposite large cushion, will also be tolerably free. Suppose the surface of the tape or large cushion to be four times greater than that of the small one, and that the pressure of this last is barely sufficient to stop the circulation in the artery, I think it must evidently appear that the degree of pressure made by the tape or large cushion, on any one point, will be only equal to the fourth part of that produced by the small one on an equal space; their surfaces being in the ratio of one to four, agreeably to my supposition; that is, supposing the pressure to be equally

 powerful powerful throughout their surfaces. Therefore, it seems reasonable to conclude, that in such a case as now mentioned, the humeral artery might be completely compressed, while, at the same time, the circulation in the fore arm and hand might be supported with nearly as great certainty as after the common operation for aneurism.

The same reasoning will apply to wounds of the extremities, attended with hæmorrhagy, which cannot be readily stopped by the usual dressings. The compressing of the principal artery or arteries that supply the part or parts with blood, from whence the hæmorrhagy takes place, undoubtedly, in most cases, will stop the bleeding; but if it should not be stopped by these means, and the proper application of dressings, a similar instrument may be applied below the wound; and thus certainly the discharge of blood would be kept within due bounds.

In many instances of wounds, it is probable that the application of these instruments, for a few hours, will be sufficient. However, the length of time must necessarily depend on the nature of the wounds. At all events, if the hæmorrhagy should recur, after the compression has been removed, either in part or entirely, the complete compression must be renewed.

The instrument alluded to is only calculated to make pressure on arteries, when they can easily be pressed against a bone by means of its cushion. On this account, I have invented another, or rather improved this instrument, by adding another semi-circle with a screw and cushion. A hinge, by which it may be readily opened and shut, is likewise added, as may be observed in fig. 5. This instrument, from its having two screws and cushions diametrically opposite, can be so managed as to compress arteries in any part of the extremities. In fact, this instrument, in a great measure, supersedes the use of the former, as one of the screws and cushion are constructed so that they may be taken away at pleasure. That part of the instrument opposite the remaining screw, may then be covered with cloth, or have a large cushion fixed to it, in order to produce an effect similar to that produced by the instrument first described.

Before concluding this subject, I consider it proper to mention, that it seems to me highly probable, that the judicious management of these instruments may be very useful in military practice, and may frequently preserve the use or even save many limbs.

Some time ago it occurred to me, that musquet balls might, might, in many instances, be extracted from wounds, by means of a steel hook, such as represented in fig. 6. The under part of the hook should form an acute angle; however, not so sharp as to injure the soft parts, and its upper part ought to be a quarter of an inch long and an eighth part in breadth, having a curvature and excavation corresponding to the shape of a musquet ball. If this instrument was cautiously introduced into a wound until it should reach the ball, it seems very probable that it might be made to pass beyond, and then turned behind the ball, in such a manner, that by a few efforts the ball could be readily extracted, namely, by withdrawing the instrument cautiously, and endeavouring at the same time to keep the hook behind, and as near its centre as possible.

I have lately invented another very simple instrument, consisting of a flat silver canula, curved at one end, together with a pliable elastic portion of steel, having likewise a similar curvature at one extremity. The whole of the steel should be of such a size as to be made to pass readily into the canula, excepting a very small portion, at its curved extremity. This portion should be constructed so as to prevent the extremity of the canula from meeting with any resistance from the soft parts when introduced into a wound. The degree of pliability of the flexible part of the steel, should be such as to render its passage through the curvature of the canula quite easy. The curved portion of steel, though inflexible, will readily pass into the curvature of the canula; their curvatures, agreeably to my supposition, being made to correspond with each other. The breadth of this instrument should be about a quarter of an inch.

Let us suppose that this instrument is placed so that its straight part forms a *tangent* to a diameter of the ball, parallel to a plane passing through the surface of the external wound, as represented in fig. 7; and that the curvature of the canula is equal in length to an eighth part of the ball's circumference (a b). Now, let us imagine, that the curved portion of steel is made to project beyond the canula for an equal distance, and it is evident that the curvatures of the canula and steel together will be equal to a fourth part of the circumference of the ball (a d). Hence I think it reasonable to suppose, that this instrument may in many cases prove extremely servicable, and will render the use of forceps frequently unnecessary.

Instead of a silver canula, it is probable that a piece of inflexible steel with groves on its edges, in order to receive the edges of the other part of the instrument, and thus to retain both portions intimately applied together, would answer better, as the instrument would be rendered thinner, and likewise more closely applied to the ball.

In fig. 8, is represented a probe made of a spiral steel wire, with a large round point. This probe may be useful in finding out the direction of sinous sores, as it readily bends in every direction, and, at the same time, possesses a sufficient degree of rigidity. When it touches a musquet ball, or any hard substance, the same kind of sensation is communicated to the hand, as when a common steel probe is rubbed against a hard substance.

## Figures and Tables

**Fig.1. Fig. 2. Fig. 3. Fig. 4. Fig.5. Fig. 6. Fig.7. Fig.8. f1:**